# A Modified Hybrid Cardioplegia Strategy Associated with Reduced Postoperative Bleeding and Re-Exploration Following Bentall Procedures: A Retrospective Single-Center Observational Study

**DOI:** 10.3390/jcdd13080353

**Published:** 2026-07-28

**Authors:** Ahmet Süha Arslan, Suat Karaca, İbrahim Özsöyler

**Affiliations:** Department of Cardiovascular Surgery, Adana City Training and Research Hospital, 01230 Adana, Türkiye; drsuatkaraca@hotmail.com (S.K.); ibrahim.ozsoyler@sbu.edu.tr (İ.Ö.)

**Keywords:** Bentall procedure, del Nido cardioplegia, blood cardioplegia, postoperative hemorrhage, re-exploration for bleeding, hemostasis, aortic root replacement

## Abstract

**Background**: Postoperative bleeding remains an important source of morbidity following Bentall procedures despite numerous technical refinements aimed at improving hemostasis. We evaluated whether a modified hybrid cardioplegia strategy incorporating intermittent blood cardioplegia during aortic root reconstruction could reduce bleeding-related complications compared with a conventional double-dose del Nido strategy. **Methods**: A total of 103 consecutive patients who underwent Bentall procedures were retrospectively analyzed. Patients were divided according to the myocardial protection strategy employed: conventional double-dose del Nido cardioplegia (Group 1, *n* = 46) and a modified hybrid strategy consisting of initial del Nido cardioplegia followed by intermittent blood cardioplegia during root reconstruction (Group 2, *n* = 57). The primary endpoint was postoperative re-exploration for bleeding during the index hospitalization. Additional bleeding-related outcomes included 24 h drainage volume and blood product transfusion requirements. Secondary endpoints were intensive care unit (ICU) stay, hospital stay, and in-hospital mortality. **Results**: Baseline characteristics, cardiopulmonary bypass times, and aortic cross-clamp durations were comparable between groups. Group 2 demonstrated significantly lower 24 h postoperative drainage volumes compared with Group 1 (450 [350–550] mL vs. 550 [400–650] mL, *p* = 0.005). Postoperative re-exploration for bleeding occurred significantly less frequently in Group 2 than in Group 1 (3.5% vs. 19.6%, *p* = 0.011). No significant differences were observed in red blood cell transfusion (*p* = 0.112), fresh frozen plasma transfusion (*p* = 0.339), or platelet transfusion requirements (*p* = 0.147). Mechanical ventilation duration (*p* = 0.423), intensive care unit stay (*p* = 0.342), and hospital stay (*p* = 0.103) were similar between groups. In-hospital mortality did not differ significantly between the groups (4.3% vs. 3.5%, *p* = 1.000); however, the study was not powered to detect differences in mortality because of the low number of events. **Conclusions**: In patients undergoing Bentall procedures, a modified hybrid cardioplegia strategy incorporating intermittent blood cardioplegia during aortic root reconstruction was associated with lower postoperative drainage volumes and fewer re-explorations for bleeding compared with a conventional double-dose del Nido strategy. These findings suggest that cardioplegia delivery may contribute to intraoperative hemostatic optimization beyond its traditional role in myocardial protection. Larger prospective studies are warranted to validate these observations.

## 1. Introduction

Composite aortic root replacement remains the standard surgical treatment for a broad spectrum of aortic root pathologies, including ascending aortic aneurysms and acute aortic dissections involving the aortic root. Ongoing refinements in operative technique and perioperative management have further improved procedural safety, durability, and clinical outcomes, establishing the Bentall procedure as the preferred option when valve-sparing approaches are not feasible [1].

Effective myocardial protection remains a fundamental component of successful aortic root surgery. The ideal cardioplegia strategy should provide reliable myocardial arrest, preserve cellular metabolism during ischemia, minimize reperfusion injury, and facilitate recovery of myocardial function following aortic cross-clamp removal. Despite decades of experience in cardiac surgery, no single cardioplegia technique has been universally accepted as the gold standard, and multiple crystalloid, blood-based, and hybrid strategies continue to be used in contemporary practice [2].

Originally developed for pediatric cardiac surgery, del Nido cardioplegia has gained widespread acceptance in adult cardiac surgery over the last decade. Large contemporary studies and recent meta-analyses have demonstrated outcomes comparable to conventional blood cardioplegia with respect to mortality and major postoperative complications while reporting potential advantages in operative workflow and myocardial preservation [3,4]. Furthermore, a recent study-level meta-analysis reported lower rates of postoperative myocardial infarction and renal failure following isolated coronary artery bypass grafting in patients receiving del Nido cardioplegia [4]. Nevertheless, concerns remain regarding prolonged ischemic periods encountered during complex aortic procedures, where supplementary myocardial reperfusion strategies may be desirable.

Several investigators have reported beneficial effects of warm blood reperfusion strategies on myocardial recovery following prolonged ischemic periods. Recent investigations have demonstrated improved heartbeat recovery, enhanced restoration of cardiac rhythm, and favorable early postoperative myocardial performance following warm blood reperfusion protocols [5,6]. Inspired by these observations, our surgical team adopted a modified cardioplegia strategy during Bentall procedures. Unlike conventional terminal warm blood cardioplegia administered immediately before aortic unclamping, intermittent blood cardioplegia was delivered following coronary button reconstruction during aortic root replacement. In addition to providing intermittent myocardial reperfusion, this approach created near-physiological pressurization at the coronary button and proximal conduit-graft anastomotic sites, enabling direct assessment of hemostasis while optimal surgical exposure was still available.

Postoperative bleeding remains one of the most important contributors to morbidity and mortality following complex cardiac surgery. Results from the VISION Cardiac Surgery study demonstrated a strong association between major postoperative complications and mortality, emphasizing the importance of preventive strategies targeting potentially modifiable perioperative risk factors [7]. In our experience, intermittent blood cardioplegia administration during aortic root reconstruction facilitated identification and correction of bleeding sites before completion of distal graft reconstruction, when surgical exposure remained optimal.

While numerous studies have compared different cardioplegia strategies with respect to myocardial protection, to our knowledge, no previous study has specifically evaluated whether blood cardioplegia administered during aortic root reconstruction can influence postoperative bleeding and the need for re-exploration for bleeding during Bentall procedures. Therefore, the present study aimed to assess the impact of a modified myocardial protection strategy, consisting of initial del Nido cardioplegia followed by blood cardioplegia during root reconstruction, on postoperative bleeding, re-exploration for bleeding, and early postoperative outcomes in patients undergoing Bentall procedures.

## 2. Materials and Methods

### 2.1. Study Design and Patient Population

This retrospective single-center observational study included consecutive adult patients who underwent Bentall procedures between January 2015 and December 2021 at our institution. The study period spanned the institutional transition from Adana Numune Training and Research Hospital to Adana City Training and Research Hospital in 2017. Following relocation, the same cardiovascular surgery department continued its clinical activities within the new institution, and patient data were retrieved from the corresponding institutional records. All patients underwent modified Bentall procedures using the coronary button reimplantation technique, and all operations were performed by the same cardiovascular surgical team using standardized operative and myocardial protection protocols.

Patient data, including demographic characteristics, perioperative variables, body mass index (BMI), and the American Society of Anesthesiologists (ASA) physical status classification, were retrospectively obtained from the institutional Hospital Information Management System (Akgün Software, Ankara, Türkiye), operative records, anesthesia charts, perfusion records, intensive care unit documentation, and hospital discharge summaries.

The study population consisted of patients undergoing composite aortic root replacement for ascending aortic aneurysm or acute aortic dissection involving the aortic root.

The choice of myocardial protection strategy was not randomized. Throughout the study period, the conventional double-dose del Nido protocol and the modified hybrid cardioplegia strategy were used concurrently rather than sequentially, with the selection largely influenced by individual surgeon preference. However, all three primary surgeons employed both cardioplegia strategies during the study period, although the relative frequency of each technique varied according to individual preference. Operative planning, myocardial protection, intraoperative hemostatic assessment, and postoperative management were performed within the same cardiovascular surgical team using standardized perioperative protocols. The distribution of cases according to the primary surgeon and cardioplegia strategy is presented in Table 1.

Because each primary surgeon employed both cardioplegia strategies, comparisons between the study groups were not exclusively attributable to surgeon-specific practice patterns. Because of the retrospective observational design of the study, no a priori sample size calculation was performed. Instead, all consecutive patients who met the predefined eligibility criteria during the study period were included, thereby representing the complete eligible institutional cohort.

#### 2.1.1. Inclusion Criteria

Patients were eligible if they:

- Were ≥18 years of age;
- Underwent Bentall procedure for elective ascending aortic aneurysm or acute aortic dissection;
- Received either conventional double-dose del Nido cardioplegia or the modified hybrid cardioplegia strategy.

#### 2.1.2. Exclusion Criteria

Patients were excluded if they:

- Underwent valve-sparing aortic root replacement;
- Required total/hemiarch replacement;
- Presented with preoperative major neurological deficits related to acute aortic dissection (stroke, coma, or severe focal neurological impairment);
- Had incomplete perioperative data.

Patients undergoing valve-sparing aortic root replacement were excluded because of their limited number and unequal distribution between study groups. Furthermore, valve-sparing root replacement differs substantially from composite graft replacement with respect to operative complexity, myocardial ischemic time, and bleeding profile. Exclusion of these patients was intended to maintain procedural homogeneity and minimize potential selection bias.

Patients requiring total/hemiarch replacement were also excluded because the substantial prolongation of cardiopulmonary bypass and aortic cross-clamp durations associated with arch reconstruction could independently influence both myocardial protection and bleeding-related outcomes, thereby confounding the evaluation of the cardioplegia strategy.

Patients presenting with preoperative major neurological deficits were excluded because these conditions are established predictors of adverse early postoperative outcomes and mortality independent of operative technique or myocardial protection strategy.

**Figure 1:**
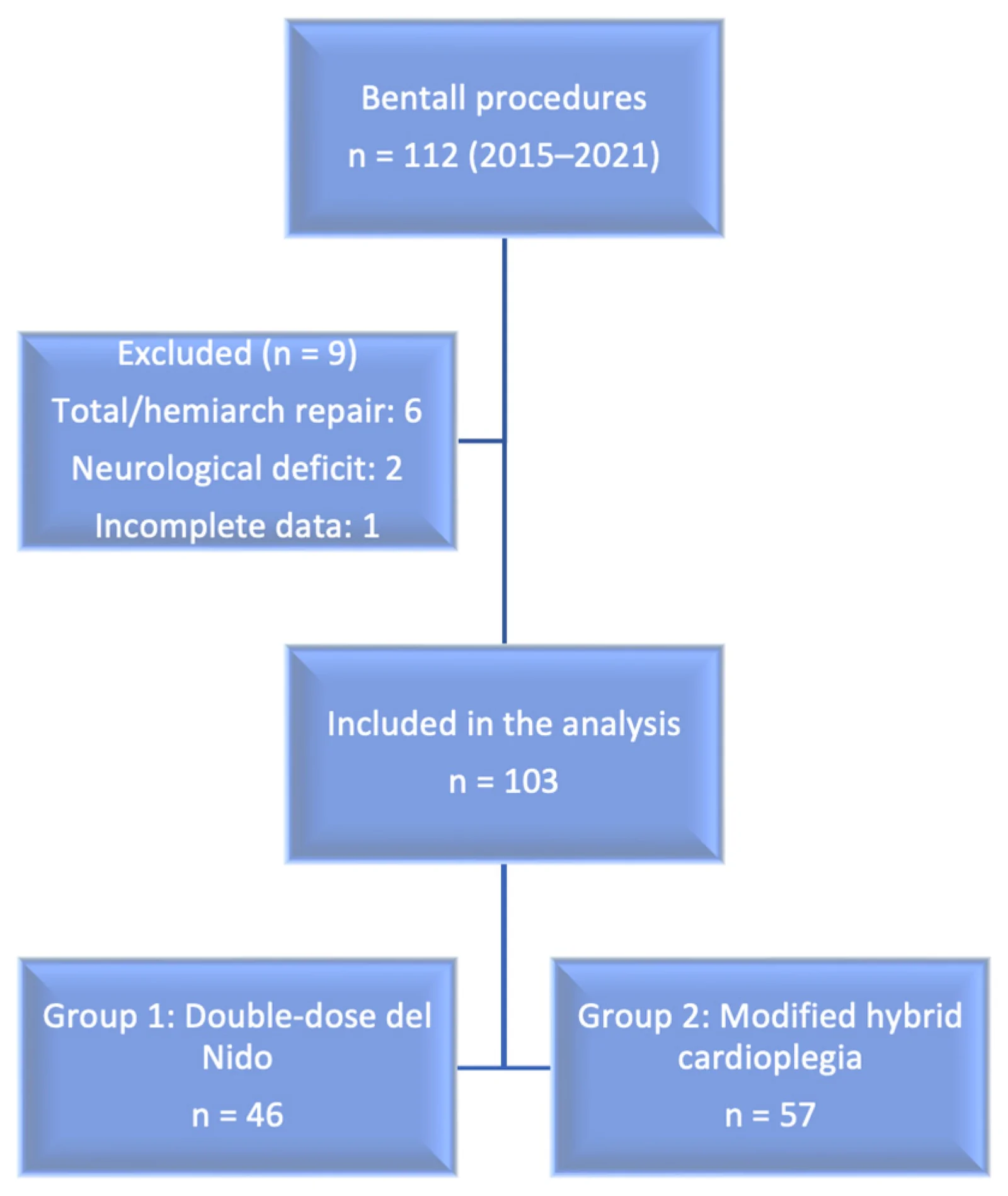
Patient flow diagram of the study cohort.

During the study period, 112 consecutive patients underwent Bentall procedures at our institution (Patient flow diagram is presented in Figure 1). Of these, 6 were excluded due to concomitant total/hemiarch replacement, 2 due to preoperative major neurological deficits, and 1 due to incomplete perioperative data.

A total of 103 patients met the eligibility criteria and were included in the final analysis.

### 2.2. Cardioplegia Protocols

#### 2.2.1. Del Nido Cardioplegia

Del Nido cardioplegia was prepared according to institutional practice using 1000 mL Plasma-Lyte A, 13 mL lidocaine 1%, 13 mL potassium chloride (2 mEq/mL), 13 mL sodium bicarbonate (8.4%), 4 mL magnesium sulfate (50%), and 16.3 mL mannitol (20%). The crystalloid solution was mixed with autologous blood in a 1:4 crystalloid-to-blood ratio and cooled to approximately 4 °C.

The initial dose was administered antegradely at 20 mL/kg, with a maximum dose of 1000 mL.

#### 2.2.2. Blood Cardioplegia

Blood cardioplegia consisted of 435 mL Ringer lactate, 20 mL mannitol (15%), 20 mL sodium bicarbonate (8.4%), and 25 mL potassium chloride (2 mEq/mL), resulting in a total crystalloid volume of 500 mL. The solution was mixed with autologous blood in a 1:4 crystalloid-to-blood ratio and delivered as tepid blood cardioplegia (24–28 °C).

#### 2.2.3. Perioperative Hemostatic Management

Perioperative anticoagulation and hemostatic management were standardized and applied consistently by the surgical team throughout the study period, irrespective of the myocardial protection strategy. Systemic anticoagulation was achieved with unfractionated heparin administered at a dose of 400 U/kg. In patients undergoing peripheral arterial cannulation, heparin was administered before cannulation. For central arterial cannulation, heparin was administered after pericardiotomy, provided that no significant pericardial adhesions were encountered. Cardiopulmonary bypass was initiated after confirmation of an activated clotting time (ACT) exceeding 400 s. If the target ACT was not achieved, additional 5000-U boluses of heparin were administered until adequate anticoagulation was achieved.

Following separation from cardiopulmonary bypass, systemic heparinization was reversed with protamine sulfate administered as a slow intravenous infusion at a 1:1 ratio to the initial heparin dose. ACT was routinely reassessed following protamine administration and transfusion of blood products, and additional protamine was administered when the post-reversal ACT remained above 150 s, indicating persistent residual heparin activity. Tranexamic acid (500 mg intravenously) was routinely administered following protamine reversal.

Topical fibrin sealant (Tisseel^®^, Baxter Healthcare, Deerfield, IL, USA) was routinely applied to the aortic root and coronary button anastomotic suture lines, as well as to the distal aortic graft anastomosis, in all patients as part of the surgical team’s standard hemostatic practice. Additional surgical hemostatic measures, such as reinforcement sutures, were performed only when clinically indicated. Point-of-care viscoelastic coagulation monitoring, including thromboelastography (TEG) and rotational thromboelastometry (ROTEM), was not routinely used during the study period. These perioperative hemostatic management practices were applied consistently by the same surgical team throughout the study period and did not differ according to the myocardial protection strategy employed.

#### 2.2.4. Thoracic Drainage Management

Thoracic drainage was managed according to the standard practice of the cardiovascular surgical team. In patients without pleural entry during median sternotomy, two 36-Fr mediastinal chest tubes were routinely placed. When one or both pleural cavities were opened, a single 36-Fr mediastinal chest tube together with one 36-Fr pleural chest tube for each opened hemithorax was inserted. All chest tubes were connected to a conventional water-seal drainage system without the routine application of external suction. Chest tubes were removed when the drainage volume decreased to <150 mL/day and no clinical or radiographic evidence of ongoing bleeding or pleural complications was present. In the present cohort, chest tubes were not routinely removed within the first 24 postoperative hours, as early postoperative mobilization was generally not feasible because of the duration of postoperative mechanical ventilation.

#### 2.2.5. Re-Exploration Criteria

The decision to perform re-exploration for postoperative bleeding was based on the standard practice of the cardiovascular surgical team and the overall clinical assessment by the cardiovascular surgical team. During the early postoperative period, excessive mediastinal drainage exceeding approximately 200 mL during the first postoperative hour or 300 mL during the second hour prompted close evaluation for possible re-exploration. Beyond the initial postoperative period, however, the decision was individualized and based on the patient’s overall clinical condition rather than chest tube output alone. Factors considered included persistent or increasing mediastinal drainage despite correction of reversible coagulopathy, progressive hemoglobin decline, ongoing packed red blood cell transfusion requirements, increasing inotropic support, hemodynamic instability (including hypotension and tachycardia), and clinical or echocardiographic suspicion of cardiac tamponade or ongoing surgical bleeding. Re-exploration was performed following consensus among the cardiovascular surgical team after integration of these clinical and laboratory findings.

### 2.3. Surgical Technique and Myocardial Protection Strategy

All procedures were performed through median sternotomy under cardiopulmonary bypass using standard institutional techniques.

All emergency procedures were performed for acute type A aortic dissection.

Patients were divided into two groups according to the myocardial protection strategy employed.

#### 2.3.1. Group 1: Conventional Double-Dose Del Nido Strategy

Following aortic cross-clamping, myocardial arrest was achieved using antegrade del Nido cardioplegia. A second dose of del Nido cardioplegia was routinely administered after 75 min of cross-clamp time. Thereafter, additional doses were administered every 60 min in cases with prolonged cross-clamp times, reflecting our surgical team’s preference for a more conservative re-dosing interval.

#### 2.3.2. Group 2: Modified Hybrid Cardioplegia Strategy

Following induction with standard del Nido cardioplegia, subsequent myocardial protection was maintained with blood cardioplegia administered at specific stages of aortic root reconstruction. After completion of the left coronary button anastomosis, the distal valved conduit graft was cross-clamped, and pressurized antegrade blood cardioplegia was administered through a needle cannula inserted into the graft, allowing near-physiological pressurization of the reconstructed aortic root for assessment of coronary button and proximal valve-graft suture-line integrity. Because the right coronary button had not yet been reimplanted at this stage, additional blood cardioplegia was selectively administered through the right coronary ostium using an ostial cardioplegia cannula to maintain myocardial protection of the right coronary territory. Following completion of the right coronary button anastomosis, blood cardioplegia was again delivered through the graft before distal graft reconstruction.

In contrast to the conventional use of terminal warm blood cardioplegia primarily for myocardial reperfusion, the purpose of this strategy extended beyond myocardial protection. Intermittent blood cardioplegia was also intended to create near-physiological pressurization of the reconstructed aortic root and coronary button anastomoses, enabling direct assessment of potential bleeding sites under optimal surgical exposure.

### 2.4. Ethical Approval

The study was approved by the Clinical Research Ethics Committee of Adana City Training and Research Hospital (Meeting Number: 108, Approval/Decision Number: 2001). The study was conducted in accordance with the principles of the Declaration of Helsinki. Owing to the retrospective design of the study, the requirement for individual informed consent was waived by the ethics committee.

### 2.5. Data Collection

The following preoperative variables were recorded:

- Age;
- Sex;
- Body mass index (BMI);
- American Society of Anesthesiologists (ASA) physical status;
- Left ventricular ejection fraction (LVEF);
- Acute aortic dissection requiring emergency surgery;
- Previous cardiac surgery (redo sternotomy);
- Preoperative hemoglobin, platelet count and International Normalized Ratio (INR).

Operative variables included:

• Cardiopulmonary bypass time;
• Aortic cross-clamp time.

### 2.6. Study Endpoints

#### 2.6.1. Primary Endpoint

The primary endpoint was postoperative re-exploration for bleeding during the index hospitalization. Late re-interventions occurring after discharge and attributable to postoperative anticoagulation-related bleeding, post-pericardiotomy syndrome, or other delayed causes were excluded from the analysis.

Additional bleeding-related outcomes included:

- 24 h chest tube drainage volume (mL);
- Packed red blood cell transfusion requirement;
- Fresh frozen plasma transfusion requirement;
- Pooled platelet transfusion requirement.

#### 2.6.2. Secondary Endpoints

Secondary endpoints included:

- Intensive care unit length of stay;
- Hospital length of stay;
- In-hospital mortality

ICU and hospital length of stay were evaluated among hospital survivors to avoid bias related to early postoperative mortality.

### 2.7. Statistical Analysis

Statistical analyses were performed using IBM SPSS Statistics for Windows, Version 25.0 (IBM Corp., Armonk, NY, USA) and Jamovi software (Version 2.3, The Jamovi Project, Sydney, Australia). Normality of continuous variables was assessed using the Shapiro–Wilk test. Normally distributed continuous variables are presented as mean ± standard deviation and were compared using Student’s t-test. Non-normally distributed continuous variables are presented as median (interquartile range) and were compared using the Mann–Whitney U test. Categorical variables are presented as number (percentage) and were compared using the Chi-square test or Fisher’s exact test, as appropriate. For the primary binary endpoint of postoperative re-exploration for bleeding, the unadjusted odds ratio (OR) and its 95% confidence interval (CI) were calculated from the corresponding 2 × 2 contingency table. All statistical tests were two-sided, and a *p* value < 0.05 was considered statistically significant.

## 3. Results

A total of 103 patients undergoing Bentall procedures were included in the study.

The annual distribution of patients receiving each cardioplegia strategy throughout the 2015–2021 study period is presented in Table 2, confirming that both strategies were used concurrently rather than in distinct chronological periods. The relatively lower number of cases in 2020 and 2021 reflects the reduced volume of elective cardiac surgical procedures during the COVID-19 pandemic; notably, the proportional distribution between the two cardioplegia strategies remained consistent with that of other years, further supporting the concurrent use of both protocols throughout the study period.

Forty-six patients received the conventional double-dose del Nido cardioplegia strategy (Group 1), whereas 57 patients received the modified hybrid cardioplegia strategy (Group 2).

Baseline demographic and preoperative characteristics are summarized in Table 3. The two groups were comparable with respect to age (55.9 ± 12.0 vs. 56.0 ± 11.2 years, *p* = 0.965), body mass index (BMI) (26.2 [24.3–29.3] vs. 27.5 [25.2–29.1] kg/m^2^, *p* = 0.374), preoperative left ventricular ejection fraction (51.2 ± 6.6% vs. 51.3 ± 6.8%, *p* = 0.925), hemoglobin level (13.8 ± 0.7 vs. 13.8 ± 0.8 g/dL, *p* = 0.653), platelet count (192 [162–226] vs. 192 [160–223] × 10^3^/µL, *p* = 0.633), and INR (1.15 ± 0.16 vs. 1.15 ± 0.16, *p* = 0.946). Male sex distribution (76.1% vs. 73.7%, *p* = 0.780), ASA physical status classification (*p* = 0.101), the incidence of emergency surgery for acute type A aortic dissection (15.2% vs. 10.5%, *p* = 0.557), and the frequency of redo surgery (6.5% vs. 3.5%, *p* = 0.654) were also similar between the two groups.

Operative characteristics are presented in Table 4. Cardiopulmonary bypass duration was comparable between the two groups (167.5 ± 39.0 vs. 167.0 ± 38.6 min, *p* = 0.412). Similarly, aortic cross-clamp times did not differ significantly (137.9 ± 32.5 vs. 130.7 ± 26.0 min, *p* = 0.170).

Primary outcome measures are shown in Table 5.

**Figure 2:**
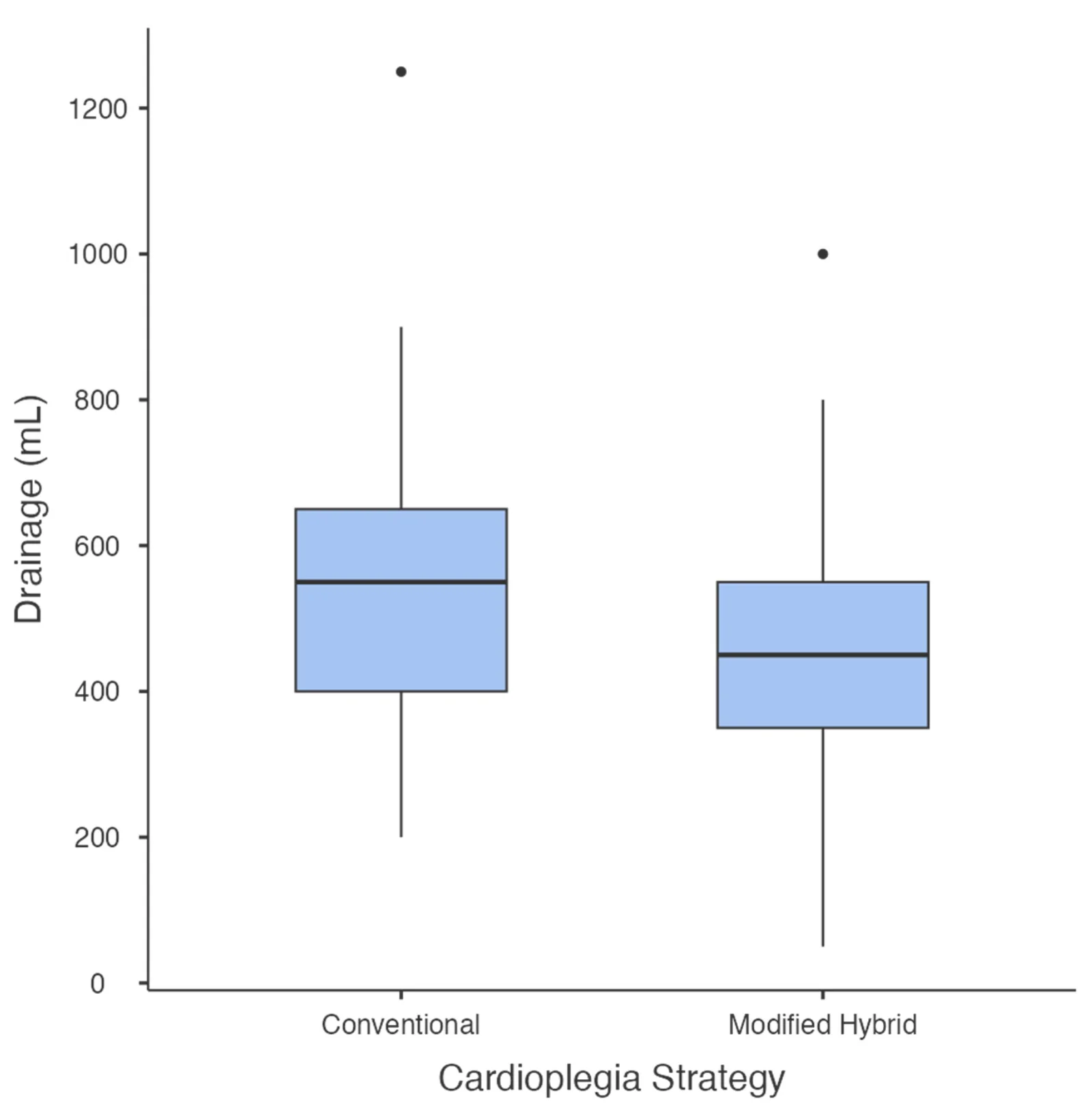
Comparison of 24 h postoperative drainage volume between the conventional double-dose del Nido and modified hybrid cardioplegia groups.

Patients in the modified hybrid cardioplegia group demonstrated significantly lower 24 h postoperative drainage volumes compared with those in the conventional double-dose del Nido group (450 [350–550] mL vs. 550 [400–650] mL, *p* = 0.005; Figure 2).

**Figure 3:**
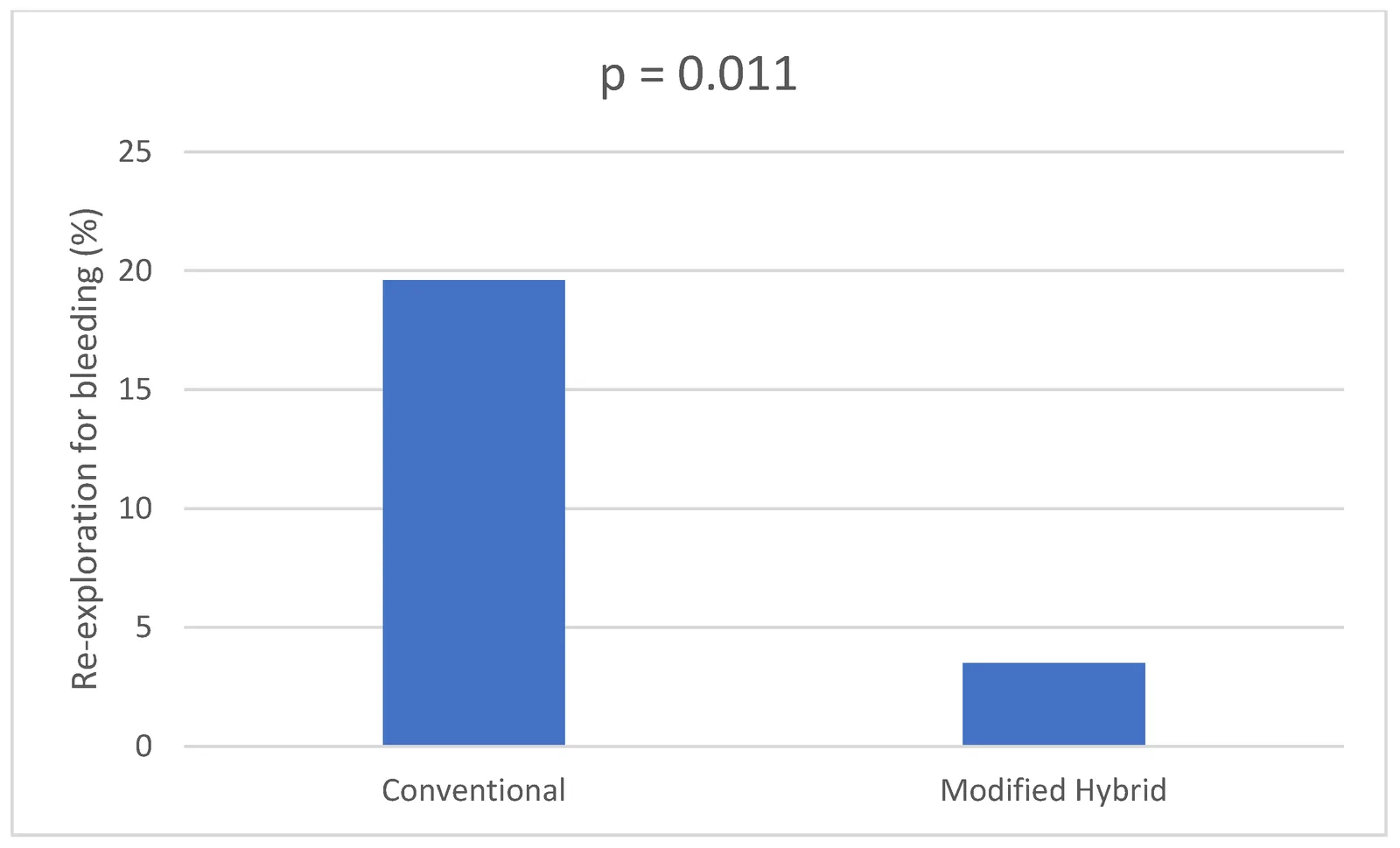
Incidence of postoperative re-exploration for bleeding according to cardioplegia strategy.

Postoperative re-exploration for bleeding occurred significantly less frequently in the modified hybrid cardioplegia group than in the conventional double-dose del Nido group (2/57 [3.5%] vs. 9/46 [19.6%]; OR, 0.15; 95% CI, 0.03–0.73; Fisher’s exact *p* = 0.011; Figure 3).

Postoperative transfusion requirements were similar between groups. Red blood cell transfusion requirements were 3 [2–4] units in Group 1 and 3 [2–3] units in Group 2 (*p* = 0.112). Fresh frozen plasma transfusion requirements were 2 [2–4] units in Group 1 and 2 [2–3] units in Group 2 (*p* = 0.339), whereas pooled platelet transfusion requirements were 0 [0–0] units in both groups (*p* = 0.147).

Secondary outcomes are summarized in Table 6. Mechanical ventilation duration was similar between groups (7 [6–8] vs. 7 [5–7.5] hours, *p* = 0.423). Likewise, ICU length of stay (2 [2–3] vs. 2 [2–2.5] days, *p* = 0.342) and total hospital length of stay (7 [6–8] vs. 6 [5–7] days, *p* = 0.103) were comparable. In-hospital mortality did not differ significantly between groups (4.3% vs. 3.5%, Fisher’s exact *p* = 1.000); however, the study was not powered to detect differences in mortality because of the low number of events.

In an exploratory analysis restricted to elective procedures, postoperative drainage volume remained significantly lower in the modified hybrid cardioplegia group (*p* = 0.037). Postoperative re-exploration for bleeding was numerically less frequent in the modified hybrid group (2.0% vs. 10.3%); however, this difference did not reach statistical significance (*p* = 0.162). Within the elective subgroup (*n* = 90), all three primary surgeons employed both cardioplegia strategies. Özsöyler performed 19 elective procedures in Group 1 and 18 in Group 2; Karaca performed 11 in Group 1 and 19 in Group 2; and Arslan performed 9 in Group 1 and 14 in Group 2. Although this exploratory subgroup analysis should be interpreted with caution because of the limited sample size and event rate, the persistence of lower postoperative drainage volumes among elective cases suggests that the observed association is unlikely to be attributable solely to differences in surgical urgency.

## 4. Discussion

The principal finding of the present study was that a modified hybrid cardioplegia strategy incorporating intermittent blood cardioplegia during aortic root reconstruction was associated with lower postoperative drainage volumes and fewer re-explorations for bleeding in patients undergoing Bentall procedures. Compared with the conventional double-dose del Nido strategy, patients receiving the modified hybrid approach demonstrated lower 24 h postoperative drainage volumes and a significantly lower incidence of postoperative re-exploration for bleeding. Importantly, these differences were observed despite comparable baseline characteristics, cardiopulmonary bypass times, and aortic cross-clamp durations between groups. Although no significant differences were observed in transfusion requirements, intensive care unit stay, hospital stay, or mortality, the modified hybrid cardioplegia strategy was associated with lower postoperative drainage volumes and fewer re-explorations for bleeding. Given the limited number of outcome events, these findings should be interpreted cautiously.

A potential explanation for the observed reduction in postoperative bleeding is the unique hemostatic advantage provided by intermittent blood cardioplegia administration during aortic root reconstruction. Importantly, the modified strategy was not designed to replace the advantages of del Nido cardioplegia during the initial phase of the operation. Del Nido cardioplegia was intentionally used to provide prolonged myocardial protection and maintain a clean, uninterrupted operative field during the technically demanding stages of aortic root dissection and coronary button reconstruction, thereby avoiding the repeated interruptions associated with frequent blood cardioplegia administration. Once the left coronary button anastomosis had been completed, blood cardioplegia was introduced to maintain myocardial protection while simultaneously allowing intraoperative hemostatic assessment.

Blood cardioplegia was subsequently delivered after completion of the left coronary button anastomosis and again following right coronary button reconstruction before distal graft anastomosis. This approach allowed pressurization of the reconstructed aortic root and graft under near-physiological pressure conditions while optimal surgical exposure was still available. As a result, potential bleeding sites originating from the coronary button anastomoses, proximal valve-graft suture line, or graft-root interface could be identified and corrected before aortic unclamping and restoration of systemic circulation. Beyond myocardial protection, this strategy transformed cardioplegia delivery into an additional intraoperative quality-control step, enabling systematic assessment of hemostasis before completion of the reconstruction. We believe that this mechanism contributed substantially to the lower postoperative drainage volumes and lower rates of re-exploration for bleeding observed in the present study. It should be noted that this proposed mechanism is inferential, based on the observed clinical outcomes and intraoperative experience of the surgical team, rather than on a systematically recorded intraoperative endpoint such as the number of additional hemostatic sutures placed during blood cardioplegia administration.

Several technical modifications have been proposed to improve hemostasis during Bentall procedures. Yakut introduced the flanged Bentall technique, in which a skirted composite graft provides a broader suture-bearing surface at the proximal anastomosis and facilitates hemostasis, particularly in fragile aortic tissues [8]. More recently, Huang et al. reported favorable early clinical outcomes using a cuff-wrapping technique based on remnant aortic root tissue, demonstrating reduced proximal anastomotic bleeding, a lower incidence of contrast extravasation, and satisfactory early postoperative results without increasing major adverse events [9]. The importance of such technical refinements has long been recognized, and previous investigators have described various hemostatic modifications aimed at reducing postoperative hemorrhage and the need for surgical re-exploration following Bentall procedures [10,11]. Collectively, these techniques share the common objective of improving hemostasis by reinforcing the reconstructed aortic root and minimizing anastomotic bleeding.

The modified hybrid cardioplegia strategy evaluated in the present study differs fundamentally from these approaches. Rather than modifying the reconstructive technique or reinforcing completed anastomoses, our strategy uses intermittent blood cardioplegia to pressurize the reconstructed aortic root before aortic unclamping, thereby allowing direct inspection of the coronary button anastomoses, proximal valve-graft suture line, and graft-root interface while surgical exposure remains optimal. This approach emphasizes prevention through early detection rather than reinforcement of an already completed reconstruction.

Interestingly, the reduction in postoperative bleeding observed in the present study persisted even after exclusion of acute type A aortic dissection cases. In an exploratory subgroup analysis restricted to elective procedures, postoperative drainage volumes remained significantly lower in the modified hybrid cardioplegia group (*p* = 0.037). Postoperative re-exploration for bleeding was also numerically less frequent in the modified hybrid group (2.0% vs. 10.3%, *p* = 0.162), although this difference did not reach statistical significance. While this subgroup analysis was not powered for clinical endpoints, the persistence of the reduction in postoperative drainage volume suggests that the observed benefit is unlikely to be attributable solely to differences in case urgency.

Among these potential bleeding sites, coronary button anastomoses deserve particular attention because they represent a technically vulnerable component of aortic root reconstruction. Coronary button anastomoses remain among the most frequent sources of bleeding following Bentall procedures. To address this problem, several technical refinements have been proposed, including felt-reinforced coronary button reconstruction and other anastomotic reinforcement techniques designed to improve hemostasis at the coronary reimplantation sites [12]. These approaches primarily focus on mechanically strengthening the completed anastomosis and reducing the risk of postoperative bleeding. In contrast, the modified hybrid cardioplegia strategy evaluated in the present study is based on early identification and correction of potential bleeding sites before restoration of systemic circulation rather than reinforcement of the completed reconstruction.

A different concept was described by Karangelis et al., who pressurized the graft with antegrade cardioplegia before right coronary button implantation to optimize coronary button positioning and minimize anastomotic tension [13]. Although the objective of that modification was not hemostatic assessment, it highlighted the practical value of evaluating the reconstructed root under pressurized conditions before completion of the operation.

Building upon this concept, the modified hybrid cardioplegia strategy evaluated in the present study systematically uses intermittent blood cardioplegia to assess the integrity of the coronary button anastomoses and proximal suture lines under physiologic pressurization. This allows immediate correction of potential bleeding sites while surgical exposure remains optimal and before restoration of systemic circulation. This mechanistic advantage is consistent with the lower postoperative drainage volumes and reduced incidence of re-exploration for bleeding observed in the present study.

Although the modified hybrid strategy was associated with significantly lower postoperative drainage volumes and fewer re-explorations for bleeding, no significant differences were observed in transfusion requirements. Several factors may explain this finding. Transfusion decisions are influenced not only by surgical bleeding but also by institutional transfusion protocols, preoperative hematologic status, hemodilution, and clinician judgment. Furthermore, blood product administration in complex aortic surgery is frequently multifactorial and may not directly parallel reductions in surgical bleeding alone. Consequently, improvements in hemostasis may be more readily reflected in drainage volumes and re-exploration rates than in transfusion requirements.

The clinical importance of preventing postoperative re-exploration extends beyond the immediate postoperative period. A recent meta-analysis including 135,456 patients demonstrated that re-exploration for bleeding was associated not only with increased operative mortality and major postoperative complications but also with significantly reduced long-term survival (HR 1.21, 95% CI 1.14–1.27) [14]. In addition, re-exploration was associated with higher risks of stroke, renal and respiratory complications, myocardial infarction, and prolonged hospital stay. These findings further emphasize that strategies capable of reducing postoperative bleeding and avoiding surgical re-exploration may have implications extending beyond early postoperative recovery.

No significant differences were observed in in-hospital mortality between the two groups (4.3% vs. 3.5%, *p* = 1.000). Given the low number of mortality events, the present study was not powered to detect differences in survival outcomes. Therefore, although the modified hybrid strategy was associated with lower postoperative drainage volumes and fewer re-explorations for bleeding, the potential impact of improved hemostasis on mortality remains uncertain and warrants evaluation in larger prospective studies.

### Study Limitations

Several limitations of the present study should be acknowledged. First, its retrospective, single-center design inherently introduces selection bias and may limit the generalizability of the findings. Second, although statistically significant differences were observed in postoperative drainage volume and re-exploration for bleeding, the reduction in re-exploration was based on only 11 outcome events and was associated with a relatively wide 95% confidence interval, reflecting the uncertainty associated with the limited number of events. Therefore, although statistically significant, the magnitude of the observed association should be interpreted with appropriate caution until confirmed in larger prospective multicenter studies. In addition, the overall sample size remained relatively modest, limiting the statistical power to detect differences in infrequent but clinically important outcomes such as mortality and other major postoperative complications. Because the decision to perform re-exploration was based on overall clinical assessment and surgical team consensus rather than on a fully standardized objective threshold, some degree of outcome misclassification cannot be excluded, although the same decision-making framework was applied consistently throughout the study period. Although the number and configuration of chest tubes may influence the total 24 h drainage volume, retrospective review of the available records and postoperative imaging did not yield sufficiently complete and standardized patient-level data on chest tube configuration. Therefore, a reliable comparison of chest tube configurations between the groups was not feasible, and residual confounding of the 24 h drainage volume analysis cannot be excluded.

Third, due to the low number of total re-exploration events (n = 11) in our cohort, advanced statistical balancing techniques, such as Propensity Score Matching (PSM) or inverse probability of treatment weighting, were not considered statistically reliable, as they would have substantially reduced the effective sample size and compromised the validity of the analyses. For the same reason, extensive multivariable regression modeling was intentionally avoided, since entering multiple covariates into a model with such a limited number of outcome events would have increased the risk of overfitting and yielded unstable estimates. Instead, we relied on univariate comparisons together with the observed similarity in baseline characteristics and operative variables to interpret our findings.

Furthermore, because of the non-randomized observational design, residual confounding cannot be completely excluded. Although the choice of myocardial protection strategy was influenced by individual surgeon preference, all three primary surgeons employed both the conventional double-dose del Nido and the modified hybrid cardioplegia strategies during the study period, albeit with different relative frequencies (Table 1). In addition, operative planning, myocardial protection, intraoperative hemostatic assessment, and postoperative management were conducted within the same cardiovascular surgical team using standardized perioperative protocols. Nevertheless, residual surgeon-related confounding cannot be entirely excluded. Moreover, the proposed mechanism by which intermittent blood cardioplegia facilitates the identification and correction of minor bleeding sites remains inferential. The available operative reports were retrospectively reviewed to determine whether additional hemostatic sutures placed during intraoperative pressurization could be reliably identified. However, because the operative reports were narrative rather than structured and did not systematically document the number, timing, or specific indication of additional reinforcement sutures, reliable patient-level extraction of this variable was not feasible. Therefore, this potential mediator could not be included in the analysis.

Finally, although an exploratory subgroup analysis restricted to elective procedures demonstrated a persistent reduction in postoperative drainage volume, the study was neither designed nor powered for definitive subgroup assessments. Accordingly, these findings remain hypothesis-generating. Larger prospective multicenter studies with standardized intraoperative hemostatic assessment and systematic documentation of additional hemostatic interventions are warranted to validate these findings and further clarify the proposed mechanism of benefit.

## 5. Conclusions

In patients undergoing Bentall procedures, a modified hybrid cardioplegia strategy incorporating intermittent blood cardioplegia during aortic root reconstruction was associated with lower postoperative drainage volumes and fewer re-explorations for bleeding compared with a conventional double-dose del Nido strategy. The proposed approach provides an opportunity for systematic intraoperative assessment of coronary button anastomoses and proximal suture lines under near-physiological pressurization before aortic unclamping. No statistically significant difference in in-hospital mortality was observed between the groups; however, the study was not powered to detect differences in mortality because of the low number of events. These findings suggest that the modified hybrid cardioplegia strategy may contribute to improved intraoperative hemostatic assessment and lower postoperative bleeding. Given the limited number of primary endpoint events, these findings should be interpreted cautiously and require confirmation in larger prospective multicenter studies.

## Figures and Tables

**Table 1:** Distribution of cases according to the primary surgeon and myocardial protection strategy.

Primary Surgeon	Group 1 (Double Dose del Nido)	Group 2 (Modified Hybrid)	Total
İbrahim Özsöyler	22	20	42
Suat Karaca	13	21	34
Ahmet Süha Arslan	11	16	27
Total	46	57	103

**Table 2:** Annual Distribution of Patients by Cardioplegia Strategy.

Year	Group 1 (del Nido) (*n* = 46)	Group 2 (Modified Hybrid) (*n* = 57)	Total (*n* = 103)
2015	5 (10.9%)	6 (10.5%)	11 (10.7%)
2016	7 (15.2%)	9 (15.8%)	16 (15.5%)
2017	8 (17.4%)	10 (17.5%)	18 (17.5%)
2018	9 (19.6%)	11 (19.3%)	20 (19.4%)
2019	8 (17.4%)	10 (17.5%)	18 (17.5%)
2020	5 (10.9%)	6 (10.5%)	11 (10.7%)
2021	4 (8.7%)	5 (8.8%)	9 (8.7%)
Total	46 (100%)	57 (100%)	103 (100%)

**Table 3:** Baseline Characteristics.

Variable	Group 1 (Conventional Double-Dose Del Nido) (*n* = 46)	Group 2 (Modified Hybrid Cardioplegia) (*n* = 57)	*p*-Value
Age (years)	55.9 ± 12.0	56.0 ± 11.2	0.965
BMI (kg/m^2^) *	26.2 [24.3–29.3]	27.5 [25.2–29.1]	0.374
Preoperative LVEF (%)	51.2 ± 6.6	51.3 ± 6.8	0.925
ASA physical status, *n* (%)			0.101
III	2 (4.3)	0 (0)	
IV	37 (80.4)	53 (93.0)	
V	7 (15.2)	4 (7.0)	
Male sex, *n* (%)	35 (76.1)	42 (73.7)	0.780
Acute Type A Dissection/Emergency Surgery, *n* (%)	7 (15.2)	6 (10.5)	0.557
Redo surgery, *n* (%)	3 (6.5)	2 (3.5)	0.654
Hemoglobin (g/dL)	13.8 ± 0.7	13.8 ± 0.8	0.653
Platelet count (×10^3^/µL) *	192 [162–226]	192 [160–223]	0.633
INR	1.15 ± 0.16	1.15 ± 0.16	0.946

* Reported as median (Q1–Q3). Comparison performed using the Mann–Whitney U test. Abbreviations: BMI, body mass index; ASA, American Society of Anesthesiologists physical status classification; LVEF, left ventricular ejection fraction; INR, international normalized ratio.

**Table 4:** Operative Characteristics.

Variable	Group 1 (*n* = 46)	Group 2 (*n* = 57)	*p*-Value
Cardiopulmonary bypass time (min)	167.5 ± 39.0	167.0 ± 38.6	0.412
Aortic cross-clamp time (min)	137.9 ± 32.5	130.7 ± 26.0	0.170

**Table 5:** Primary Outcomes.

Variable	Group 1	Group 2	*p*-Value
24 h drainage (mL)	550 [400–650]	450 [350–550]	**0.005**
RBC transfusion (units)	3 [2–4]	3 [2–3]	0.112
FFP transfusion (units)	2 [2–4]	2 [2–3]	0.339
Pooled platelet transfusion (units)	0 [0–0]	0 [0–0]	0.147
Re-exploration for bleeding, *n* (%)	9 (19.6)	2 (3.5)	**0.011**

Continuous variables are presented as median (Q1–Q3) and compared using the Mann–Whitney U test. Re-exploration for bleeding is presented as *n* (%) and compared using Fisher’s exact test. Bold values indicate statistical significance at *p* < 0.05.

**Table 6:** Secondary Outcomes.

Variable	Group 1	Group 2	*p*-Value
Mechanical ventilation time (hours) *	7 [6–8]	7 [5–7.5]	0.423
ICU stay (days) *	2 [2–3]	2 [2–2.5]	0.342
Hospital stay (days) *	7 [6–8]	6 [5–7]	0.103
In-hospital mortality, n (%)	2 (4.3)	2 (3.5)	1.000

* Continuous variables are reported as median (Q1–Q3) and compared using the Mann–Whitney U test.

## Data Availability

The datasets generated and/or analyzed during the current study are not publicly available due to patient privacy considerations but are available from the corresponding author upon reasonable request and with permission of the relevant institutional authorities.

## Abbreviations

The following abbreviations are used in this manuscript:

ICU	Intensive Care Unit
BMI	Body Mass Index
ASA	American Society of Anesthesiologists physical status classification
INR	International Normalized Ratio
LVEF	Left Ventricular Ejection Fraction
RBC	Red Blood Cell
FFP	Fresh Frozen Plasma

## References

[1] Isselbacher E.M., Preventza O., Hamilton Black J., Augoustides J.G., Beck A.W., Bolen M.A., Braverman A.C., Bray B.E., Brown-Zimmerman M.M., Chen E.P. (2022). 2022 ACC/AHA Guideline for the Diagnosis and Management of Aortic Disease: A Report of the American Heart Association/American College of Cardiology Joint Committee on Clinical Practice Guidelines. Circulation.

[2] Li C., Xiang H., Yang H., Liu W., Lan W., Luo C., Han S., Li Y., Tang Y. (2024). Del Nido cardioplegia versus cold blood cardioplegia in adult cardiac surgery: A meta-analysis of randomized clinical trials. J. Cardiothorac. Surg..

[3] Hawkins R.B., Stewart J.W., Wu X., Goldberg J., Fitzgerald D., DeLucia A., Graebner B., Willekes C., Pagani F.D., Nieter D.H. (2024). Del Nido versus blood cardioplegia in cardiac surgery: A multicenter analysis of over 40,000 patients. J. Thorac. Cardiovasc Surg..

[4] Yamashita Y., Baudo M., Magouliotis D.E., Sicouri S., Wertan M.A.C., Spragan D.D., Torregrossa G., Ramlawi B., Sutter F.P. (2025). Effect of del Nido Cardioplegia on Isolated Coronary Artery Bypass Grafting: A Study-level Meta-analysis. J. Cardiothorac. Vasc. Anesth..

[5] Satdhabudha O., Songvasin M., Homvises B., Noppawinyoowong N., Chanawangsa P., Kaewbunjong J. (2024). Can heartbeat recovery be improved with terminal non-cardioplegic warm blood perfusion prior to aortic unclamping in single-clamp technique coronary artery bypass surgery? A randomized controlled trial. J. Cardiothorac. Surg..

[6] Totaro P., Musto M., Tulumello E., Degani A., Argano V., Pelenghi S. (2025). Selective Vein Graft Cold Cardioplegia and Warm Reperfusion to Enhance Early Recovery in Patients with Left Ventricle Depression Undergoing Coronary Artery Surgery. J. Cardiovasc Dev. Dis..

[7] Lamy A., Browne A., Whitlock R.P., Chan M.T.V., Underwood M.J., Parlow J.L., Allard R.V., Lomivorotov V.V., Marat A., Landoni G. (2026). Association between complications and mortality after cardiac surgery: Results from the VISION Cardiac Surgery prospective cohort study. J. Thorac. Cardiovasc Surg..

[8] Yakut C. (2001). A new modified Bentall procedure: The flanged technique. Ann. Thorac. Surg..

[9] Huang L.C., Liu C., Fan S.Y., Sun Y.X., Guo H.W. (2023). Clinical outcomes of the cuff wrapping technique in the modified Bentall procedure: A propensity score-matched study. J. Thorac. Dis..

[10] Della Corte A., Baldascino F., La Marca F., Scardone M., Nappi G., Cefarelli M., De Santo L.S., Pepino P., Cotrufo M., De Feo M. (2012). Hemostatic modifications of the Bentall procedure: Imbricated proximal suture and fibrin sealant reduce postoperative morbidity and mortality rates. Tex. Heart Inst. J..

[11] Copeland J.G., Rosado L.J., Snyder S.L. (1993). New technique for improving hemostasis in aortic root replacement with composite graft. Ann. Thorac. Surg..

[12] Akkaya Ö., Jalalzai I., Arslan Ü. (2026). Posterior Teflon-Felt-Reinforced Coronary Button Anastomosis in a Modified Bentall Procedure: Early Outcomes in a Single-Center Retrospective Study. J. Clin. Med..

[13] Karangelis D., Tzertzemelis D., Demis A.A., Economidou S., Panagiotou M. (2018). Eighteen years of clinical experience with a modification of the Bentall button technique for total root replacement. J. Thorac. Dis..

[14] Soletti G., Cancelli G., Dell’Aquila M., Caldonazo T., Harik L., Rossi C., Tasoudis P., Leith J., An K.R., Dimagli A. (2024). Re-exploration for bleeding and long-term survival after adult cardiac surgery: A meta-analysis of reconstructed time-to-event data. Int. J. Surg..

